# Relationship between Aging-Related Skin Dryness and Aquaporins

**DOI:** 10.3390/ijms18071559

**Published:** 2017-07-18

**Authors:** Nobutomo Ikarashi, Risako Kon, Miho Kaneko, Nanaho Mizukami, Yoshiki Kusunoki, Kiyoshi Sugiyama

**Affiliations:** 1 Department of Clinical Pharmacokinetics, Hoshi University, 2-4-41 Ebara, Shinagawa-ku, Tokyo 142-8501, Japan; m1703@hoshi.ac.jp (M.K.); m1616@hoshi.ac.jp (N.M.); p16kusunoki@hoshi.ac.jp (Y.K.); sugiyama@hoshi.ac.jp (K.S.); 2 Global Research Center for Innovative Life Science, Hoshi University, 2-4-41 Ebara, Shinagawa-ku, Tokyo 142-8501, Japan; r-kon@hoshi.ac.jp

**Keywords:** aquaporin, skin, dermal water content, aged, young

## Abstract

Skin function deteriorates with aging, and the dermal water content decreases. In this study, we have analyzed the mechanism of aging-related skin dryness focusing on aquaporins (AQPs), which are the water channels. Mice aged 3 and 20 months were designated as young and aged mice, respectively, to be used in the experiments. No differences were observed in transepidermal water loss between the young mice and aged mice. However, the dermal water content in aged mice was significantly lower than that in young mice, thus showing skin dryness. The expression of AQP1, AQP3, AQP4, AQP7, and AQP9 was observed in the skin. All the mRNA expression levels of these AQPs were significantly lower in aged mice. For AQP3, which was expressed dominantly in the skin, the protein level was lower in aged mice than in young mice. The results of the study showed that the expression level of AQPs in the skin decreased with aging, suggesting the possibility that this was one of the causes of skin dryness. New targets for the prevention and treatment of aging-related skin dryness are expected to be proposed when the substance that increases the expression of AQP3 is found.

## 1. Introduction

Aquaporins (AQPs) are a family of water channels that transport water and small molecules for the maintenance of fluid homeostasis. AQPs are present in many organisms, including bacteria and humans. Thirteen AQP family members have been identified in humans (AQP0-12), and are expressed in various organs [1]. Abnormalities in the expression level of AQPs have recently been found to cause various diseases. For example, it has become clear that nephrogenic diabetes insipidus occurs when the expression level of AQP2 in the kidney decreases [2], cerebral edema occurs when the expression level of AQP4 in the brain increases [3,4], constipation and diarrhea are induced when the expression level of AQP3 in the large intestine increases or decreases [5,6,7]. Therefore, control of the expression level of AQPs may be effective for the prevention and treatment of various diseases.

AQP3 is abundantly expressed in human and mouse skin epidermis [8,9]. Because the water content in the stratum corneum of epidermis is reduced in AQP3-null mice, it is thought that AQP3 plays a key role in skin hydration [10,11]. AQP3-null mice exhibit delayed barrier recovery after acute barrier disruption [12]. In contrast, transgenic mice overexpressing AQP3 exhibit accelerated barrier recovery [13]. AQP3 also facilitates migration and proliferation of keratinocytes in epidermis during wound healing [14]. Thus, AQPs in the skin have various functions [15].

It is known that skin function deteriorates with aging, and that this is associated with a decrease in the dermal water content [16]. It has also been reported that this decrease in the dermal water content involves a decrease in hyaluronic acid, ceramide, and collagen amounts in the skin [17,18,19]. However, although there are cosmetics and health foods that target hyaluronic acid and collagen, there are few products that improve aging-related skin dryness. The aim of this study was to investigate the possibility that AQPs may serve as new target molecules for aging-related skin dryness. Briefly, mice aged 3 and 20 months were designated as young and aged mice, respectively, to analyze the expression level of AQPs in the skin.

## 2. Results

### 2.1. Body Weight, Transepidermal Water Loss (TEWL) and Dermal Water Content

Body weight, TEWL, and dermal water content were measured in mice aged 3 (young mice) and 20 months (aged mice).

**Figure 1:**
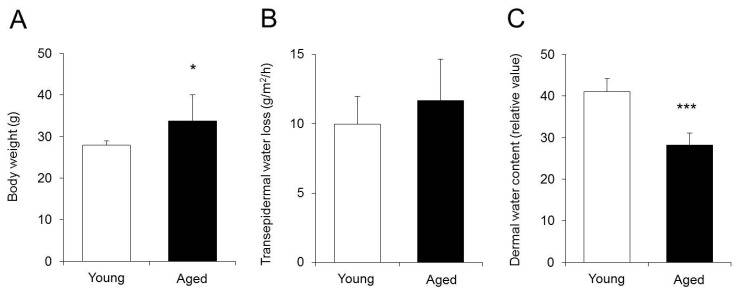
Body weight, transepidermal water loss (TEWL), and dermal water content. Young and aged mice had their body weight (**A**); TEWL (**B**); and dermal water content (**C**) measured. The data represent the means ± SDs for five mice. Student’s *t*-test: * *p* < 0.05 and *** *p* < 0.001 vs. Young mice.

Body weight was significantly increased in aged mice compared to that in young mice (Figure 1A). TEWL was approximately the same in aged and young mice, showing no significant difference (Figure 1B). The dermal water content was significantly lower in aged mice than in young mice (Figure 1C).

Based on the above, it was found that the dermal water content in aged mice did not decrease because of increased TEWL.

### 2.2. The Expression Level of Aquaporins (AQPs) in the Skin

The expression level and distribution of AQPs in the skin of young and aged mice were analyzed.

**Figure 2:**
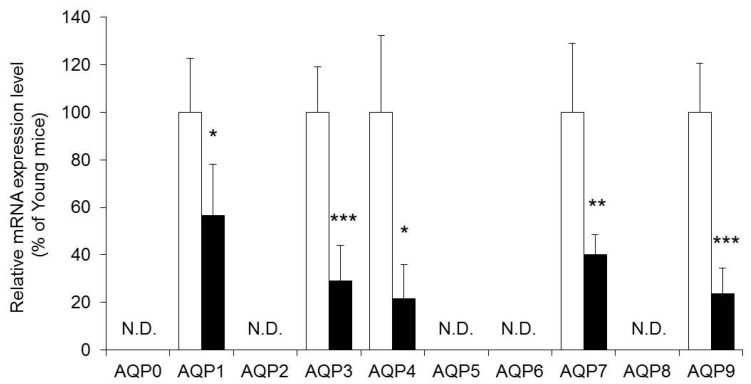
The mRNA expression level of aquaporins (AQPs) in the skin. The whole skin, containing epidermis and dermis, was harvested from young (white) and aged mice (black). The mRNA expression level of AQPs in the skin were measured using real-time RT-PCR, and normalized to β-actin. Mean levels of AQP mRNA expression of young mice are indicated as 100%. The data represent the means ± SDs for five mice. Student’s *t*-test: * *p* < 0.05, ** *p* < 0.01 and *** *p* < 0.001 vs. Young mice. N.D.: Not detect.

Among the AQP0 through AQP9 analyzed in this study, the expression of AQP1, AQP3, AQP4, AQP7, and AQP9 was confirmed in the skin of HR-1 hairless mice. All the mRNA expression levels of these AQPs were significantly lower in aged mice than in young mice (Figure 2).

**Figure 3:**
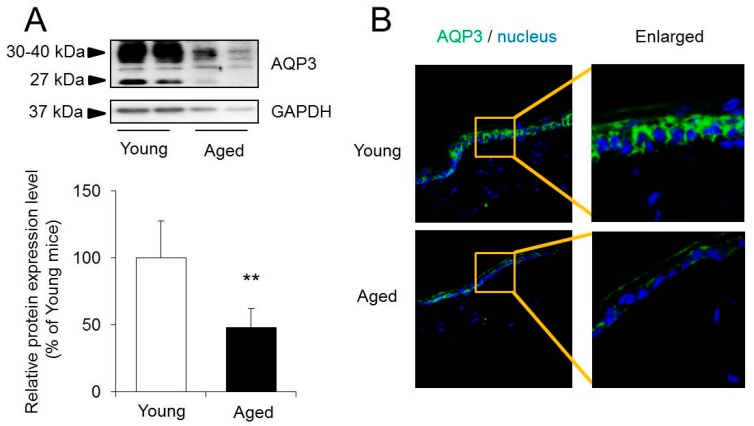
The protein expression level of AQP3 in the skin. The whole skin, containing epidermis and dermis, was harvested from young and aged mice. (**A**) The protein expression level of AQP3 in the skin was measured using western blotting, and normalized to glyceraldehyde-3-phosphate dehydrogenase (GAPDH). Mean levels of AQP3 protein expression of young mice were indicated as 100%. The data represent the means ± SDs for five mice. Student’s *t*-test: ** *p* < 0.01 vs. Young mice. (**B**) AQP3 (green) and nuclei (blue) were immunostained.

The protein expression level was analyzed for AQP3, which is known to be closely related to moisture retention in the skin [10,11]. In western blotting, the bands of AQP3 were detected at 27 kDa and 30 to 40 kDa. One of these bands appeared at approximately 27 kDa and represented the deglycosylated form of AQP3. The other appeared at approximately 30 to 40 kDa and represented a glycosylated form of AQP3 [20,21]. Glycosylation was associated with the stability and cell surface expression of AQPs, but it does not influence water permeability [22,23,24]. In this study, the sum of these bands was analyzed as the expression level of AQP3. The protein expression level of AQP3 in the skin was significantly lower in aged than in young mice (Figure 3A). When the whole skin, containing epidermis and dermis, was harvested from the mice, AQP3 was only detected in the stratum basal (Figure 3B) [8]. In the immunohistochemistry, it was also confirmed that the density of AQP3 in the skin of aged mice was lower than that of young mice (Figure 3B).

Based on the above, it was found that the expression level of skin AQPs decreased in aged mice compared to that in young mice.

### 2.3. The Expression Level of AQPs in the Whole Body

**Figure 4:**
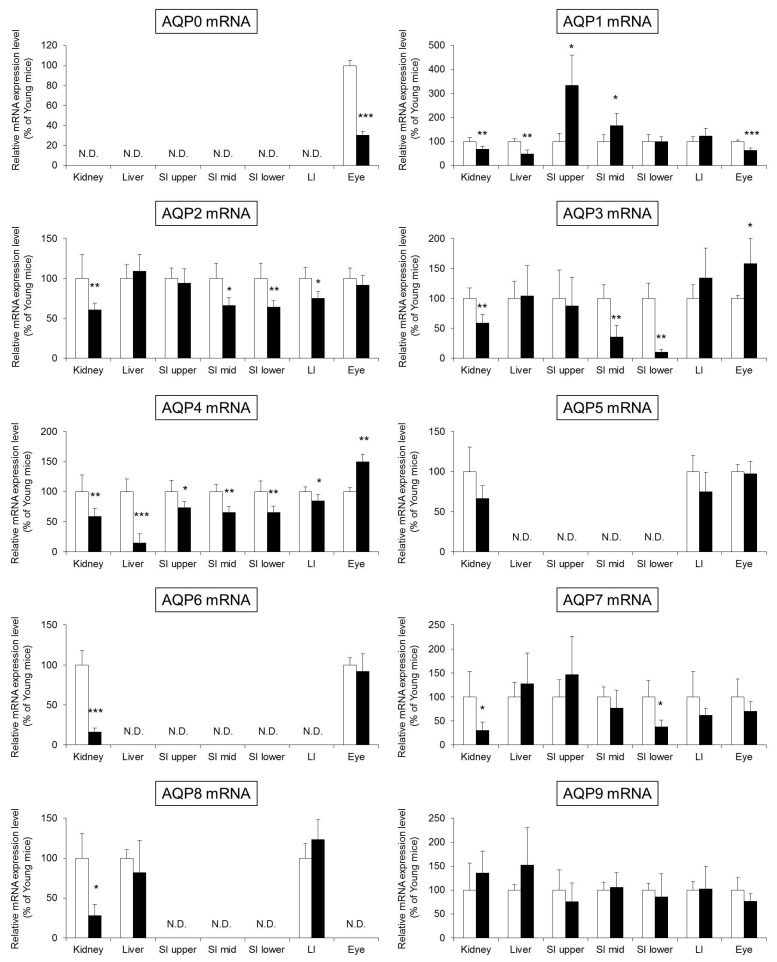
The expression level of AQPs in the whole body. The kidney, liver, small intestine (SI), large intestine (LI), and eye were harvested from young (white) and aged mice (black). The mRNA expression level of AQPs were measured using real-time RT-PCR and normalized to β-actin. Mean levels of AQPs mRNA expression of young mice are indicated as 100%. The data represent the means ± SDs for five mice. Student’s *t*-test: * *p* < 0.05, ** *p* < 0.01, and *** *p* < 0.001 vs. Young mice. N.D.: Not detect.

The results showed that the expression level of various members of the AQPs family in the skin decreased along with aging. We investigated whether the aging-related decreased AQPs expression was specific to the skin or if it also changed in the other organs. Briefly, the mRNA expression level of AQPs was analyzed in the kidney, liver, upper small intestine, middle small intestine, lower small intestine, large intestine, and eye of young and aged mice (Figure 4).

The mRNA expression of AQP0 was observed only in the eye, and the expression level of aged mice was significantly lower than that in young mice by approximately 40%.

The expression level of AQP1 was significantly lower in aged mice than in young mice in the kidney, liver, and eye. On the other hand, the expression level of AQP1 in the upper and middle small intestine was significantly higher in aged mice than in young mice.

The expression level of AQP2 in the kidney, middle small intestine, lower small intestine, and large intestine was significantly lower in aged mice than in young mice.

The expression level of AQP3 in the kidney, middle small intestine, and lower small intestine was significantly lower in aged mice than in young mice. On the other hand, the expression level of AQP3 in the eye was significantly higher in aged mice than in young mice.

The expression level of AQP4 in the kidney, liver, small intestine, and large intestine was significantly lower in aged mice than in young mice. On the other hand, the expression level of AQP4 in the eye was significantly higher in aged mice than in young mice.

The expression of AQP5 was observed in the kidney, large intestine, and eye, with no differences between young and aged mice.

The expression of AQP6 was observed in the kidney and eye. The expression level of AQP6 in the kidney was significantly lower in aged mice than in young mice.

The expression level of AQP7 in the kidney and lower small intestine was significantly lower in aged mice than in young mice.

The expression of AQP8 was observed in the kidney, liver, and large intestine. The expression level of AQP8 in the kidney was significantly lower in aged mice than in young mice.

The expression level of AQP9 in any of the analyzed organs showed no differences between young and aged mice.

Based on the above, it was found that the aging-related change in the expression of AQPs differed significantly depending on the organ and the type of AQP.

## 3. Discussion

It is known that a decrease in the amount of hyaluronic acid, ceramide, and collagen is involved in the reduction of skin function, particularly aging-related skin dryness [16]. Recently, many cosmetics and health foods that target these have been developed as people are becoming more conscious of anti-aging. However, no substances that are dramatically effective for aging-related skin dryness have been found, and it is considered important to clarify the mechanism of onset of this condition and to develop new measures. This study aimed to clarify the role of AQPs in aging-related skin dryness, and to propose the possibility that AQPs may serve as target molecules for aging-related skin dryness. In this study, mice aged 3 and 20 months were designated as young and aged mice, respectively, to be used in the experiment. Aged mice were assumed to represent 60 years of age in humans based on the lifespans of mice and humans [25].

There was no difference in TEWL between young and aged mice (Figure 1B). In contrast, the dermal water content was significantly lower in aged mice than in young mice (Figure 1C). Normally, water is transported in the skin from the vessel side to the corneum side and is retained in the corneum. The dermal water content decreases when the barrier function of the corneum is disrupted and TEWL increases [26]. The results of this study showed that skin dryness due to aging was not caused by decreased barrier function.

The cause of the decrease in the dermal water content was analyzed focusing on AQPs in the skin. The expression level of AQP3, which was dominantly expressed in the skin, and plays an important role in the maintenance of the water content [10,11], was significantly lower in aged mice than in young mice both based on the mRNA level and the protein level (Figure 2 and Figure 3). Decreased expression of AQP3 has been determined to be one of the factors involved in skin dryness in aged mice. It has been reported that AQP3 also affects the migration capacity and growth of keratinocytes [14]. It was therefore considered possible that the aging-related decreased expression of skin AQP3 was not only involved in a decrease in the dermal water content, but also in the aging-related delay of wound healing.

This study also showed that AQP1, AQP4, AQP7, and AQP9 were expressed in the mouse skin in addition to AQP3, and all of these AQPs were significantly lower in aged mice than in young mice (Figure 2). AQP1, AQP3, AQP5, AQP7, AQP9, and AQP10 are observed to be expressed in human skin [8]. AQP5 is expressed in the sweat glands and is involved in the secretion of sweat [27]. It was therefore possible that a decreased expression of AQP5 was involved in aging-related abnormal sweat secretion. AQP1 is expressed in the melanocytes [8]. Melanocytes synthesize melanin, and are involved in skin pigmentation and defense against radiation. Little is known about how much AQP1 contributes to water transportation in the skin, but it cannot be denied that the aging-related decreased expression of AQP1 may cause senile pigment freckles, leukoderma senile, etc., because the AQP family has various functions. AQP9 is expressed in keratinocytes and Langerhans cells, and AQP7 is expressed in adipocytes [8], but little is known about their function in the skin. AQP7 and AQP9 are involved in cell motility similar to the other members of the AQPs family, and therefore, it is possible that the aging-related decreased expression of AQP7 and AQP9 may affect delayed wound healing [28,29,30]. It is possible that a new therapeutic strategy can be developed for skin diseases when the function of these AQPs in the skin is clarified in the future [15].

We investigated whether the aging-related decreased expression of AQPs also shows any changes in organs other than the skin. The expression levels of AQP2 and AQP3 in the kidney have been found to decrease with aging, and it is suggested that this may be involved in abnormalities of urinary excretion in elderly people [31]. It has also been reported that the expression level of AQP0 in the eye decreases with aging, and this has been shown to be involved in aging-related decrease in the visual function [32]. As described above, based on the results of studies on aging in various functional molecules, including AQPs, it was predicted that the expression level of AQPs would decrease with aging in all organs. However, the results of the present analysis showed that there were AQPs that increased significantly, including AQP1 in the small intestine and AQP3 in the eye (Figure 4). Although the mechanism of changes in the expression of these AQPs and their role is unknown, these data provide an important explanation of aging-related water metabolism abnormalities in various organs. Moreover, the results of comprehensive analysis of the AQP family in various organs of the same individual are also useful observations to estimate the water balance between organs.

The results of the present study showed that the expression level of AQPs in the skin decreased with aging, and suggested the possibility that this was one of the causes of aging-related skin dryness. Recently, substances that increase the expression level of AQPs in the skin have been actively sought out [33,34,35]. It is possible that AQPs may serve as target molecules for the prevention and treatment of senile xerosis by clarifying the effects of these substances on aging-related skin dryness.

## 4. Materials and Methods

### 4.1. Animals and Treatments

The animal experiments were performed in accordance with the protocol approved by the Committee on the Ethics of Animal Experiments of Hoshi University (09-72, 4 November 2009). The mice were kept at room temperature (24 ± 1 °C) at 55 ± 5% humidity with a 12-h light cycle (artificial illumination: 08:00–20:00). HR-1 hairless mice (2 months old) were purchased from Hoshino Laboratory Animals, Corp. (Ibaraki, Japan). After breeding up to 3 or 20 months old, the skin, kidney, liver, small intestine, large intestine, and eye were removed, frozen in liquid nitrogen, and stored at −80 °C. All surgery was performed under diethyl ether anesthesia, and all efforts were made to minimize suffering.

### 4.2. Measurement of TEWL and Dermal Water Content

TEWL was measured using a Tewameter (TM300, Courage & Khazaka, Cologne, Germany). The dermal water content was measured using a Corneometer (CM825, Courage & Khazaka, Cologne, Germany). These measurements were carried out at 23 ± 1 °C and 60 ± 10% humidity.

### 4.3. Real-Time RT-PCR

RNA was extracted from the mouse tissue using TRI reagent (Sigma-Aldrich Corp., St. Louis, MO, USA). A high-capacity cDNA synthesis kit (Applied Biosystems, Foster City, CA, USA) was used to synthesize cDNA from 1 μg of RNA. Target gene expression was analyzed with real-time RT-PCR using the primers listed in Table 1. The mRNA gene expression levels were normalized to β-actin gene expression levels.

### 4.4. Preparation of Membrane Fraction from Skin for Immunoblotting

The skin tissue was homogenized in a dissecting buffer (0.3 M sucrose, 25 mM imidazole, 1 mM ethylenediaminetetraacetic acid, 8.5 µM leupeptin, 1 µM phenylmethylsulfonyl fluoride; pH 7.2) using a Physcotron homogenizer on ice. The homogenate was centrifuged (4000× *g* for 15 min at 4 °C), and the resulting supernatant was centrifuged (200,000× *g* for 60 min at 4 °C). The supernatant was removed, and dissecting buffer was added to the precipitate. The final homogenate contained the crude membrane fraction.

### 4.5. Electrophoresis and Immunoblotting

The proteins were separated using sodium dodecyl sulfate polyacrylamide gel electrophoresis, and then were transferred to a polyvinylidene difluoride membrane. The protein in the membrane was reacted with the primary antibodies and secondary antibodies. The protein was detected using the electrochemiluminescence prime detection reagent (GE Healthcare, Chalfont St. Giles, UK). Proteins were visualized using an LAS-3000 Mini Lumino image analyzer (Fuji Film, Tokyo, Japan). The protein expression level of AQP3 was normalized to glyceraldehyde-3-phosphate dehydrogenase (GAPDH).

### 4.6. Immunohistochemistry

The whole skin, containing epidermis and dermis, was post-fixed in 4% paraformaldehyde in phosphate-buffered saline (PBS). The samples were embedded, and the frozen blocks were cut using a cryostat (Leica Microsystems, Tokyo, Japan). The sections were reacted with a rabbit anti-rat AQP3 antibody (Alomone Labs, Jerusalem, Israel). The sections were treated with an Alexa Fluor 488 donkey anti-rabbit IgG antibody (Invitrogen Corp., Tokyo, Japan) and a 4′,6-diamidino-2-phenylindole solution (Wako Pure Chemicals, Osaka, Japan). The immunostained sections were observed under a fluorescent microscope (FV1200 IX83, Olympus Corporation, Tokyo, Japan).

### 4.7. Statistical Analyses

Numerical data were expressed as mean ± standard deviation. Significance was examined using Student’s *t*-test for pairs of values. Differences of *p* < 0.05 were considered to be statistically significant.

## Figures and Tables

**Table 1:** Primer sequences for real-time PCR.

Gene	Forward	Reverse
AQP0	TGCTCTGCATCTTTGCTACA	GCACCAGTGTAATACATCCCA
AQP1	CTGCTGGCGATTGACTACACT	TCATAGATGAGCACTGCCAGG
AQP2	CTGGCTGTCAATGCTCTCCAC	TTGTCACTGCGGCGCTCATC
AQP3	CCTTGTGATGTTTGGCTGTGG	GGAAGCACATTGCGAAGGTC
AQP4	GAGTCACCACGGTTCATGGA	CGTTTGGAATCACAGCTGGC
AQP5	GCTGGAGAGGCAGCATTG	CACCCAAGTGTCCCATCATG
AQP7	GCTTGGTCTGCTGCTTCAG	GGAACTCTGCCAGAAACTCTC
AQP8	GGCTTCTCTGTCATTGTGGA	TCCGATGAGGAGCCTAATGA
AQP9	TGAGCCATTAGGAGAGACCTT	ACCTCCAACTTTAGTCCACCA
β-actin	GAGCGCAAGTACTCTGTGTG	CGGACTCATCGTACTCCTG

## Abbreviations

AQPs	aquaporins
TEWL	transepidermal water loss
PBS	phosphate-buffered saline

## References

[1] Ishibashi K., Hara S., Kondo S. (2009). Aquaporin water channels in mammals. Clin. Exp. Nephrol..

[2] Rojek A., Fuchtbauer E.M., Kwon T.H., Frokiaer J., Nielsen S. (2006). Severe urinary concentrating defect in renal collecting duct-selective AQP2 conditional-knockout mice. Proc. Natl. Acad. Sci. USA.

[3] Binder D.K., Oshio K., Ma T., Verkman A.S., Manley G.T. (2004). Increased seizure threshold in mice lacking aquaporin-4 water channels. Neuroreport.

[4] Manley G.T., Binder D.K., Papadopoulos M.C., Verkman A.S. (2004). New insights into water transport and edema in the central nervous system from phenotype analysis of aquaporin-4 null mice. Neuroscience.

[5] Ikarashi N., Baba K., Ushiki T., Kon R., Mimura A., Toda T., Ishii M., Ochiai W., Sugiyama K. (2011). The laxative effect of bisacodyl is attributable to decreased aquaporin-3 expression in the colon induced by increased PGE2 secretion from macrophages. Am. J. Physiol. Gastrointest. Liver Physiol..

[6] Kon R., Ikarashi N., Hayakawa A., Haga Y., Fueki A., Kusunoki Y., Tajima M., Ochiai W., Machida Y., Sugiyama K. (2015). Morphine-Induced Constipation Develops With Increased Aquaporin-3 Expression in the Colon via Increased Serotonin Secretion. Toxicol. Sci. Off. J. Soc. Toxicol..

[7] Kon R., Ikarashi N., Nagoya C., Takayama T., Kusunoki Y., Ishii M., Ueda H., Ochiai W., Machida Y., Sugita K. (2014). Rheinanthrone, a metabolite of sennoside A, triggers macrophage activation to decrease aquaporin-3 expression in the colon, causing the laxative effect of rhubarb extract. J. Ethnopharmacol..

[8] Boury-Jamot M., Sougrat R., Tailhardat M., Le Varlet B., Bonte F., Dumas M., Verbavatz J.M. (2006). Expression and function of aquaporins in human skin: Is aquaporin-3 just a glycerol transporter?. Biochim. Biophys. Acta.

[9] Sougrat R., Morand M., Gondran C., Barre P., Gobin R., Bonte F., Dumas M., Verbavatz J.M. (2002). Functional expression of AQP3 in human skin epidermis and reconstructed epidermis. J. Investig. Dermatol..

[10] Hara-Chikuma M., Verkman A.S. (2005). Aquaporin-3 functions as a glycerol transporter in mammalian skin. Biol. Cell.

[11] Ma T., Hara M., Sougrat R., Verbavatz J.M., Verkman A.S. (2002). Impaired stratum corneum hydration in mice lacking epidermal water channel aquaporin-3. J. Biol. Chem..

[12] Hara M., Ma T., Verkman A.S. (2002). Selectively reduced glycerol in skin of aquaporin-3-deficient mice may account for impaired skin hydration, elasticity, and barrier recovery. J. Biol. Chem..

[13] Qin H., Zheng X., Zhong X., Shetty A.K., Elias P.M., Bollag W.B. (2011). Aquaporin-3 in keratinocytes and skin: Its role and interaction with phospholipase D2. Arch. Biochem. Biophys..

[14] Hara-Chikuma M., Verkman A.S. (2008). Aquaporin-3 facilitates epidermal cell migration and proliferation during wound healing. J. Mol. Med..

[15] Patel R., Kevin Heard L., Chen X., Bollag W.B. (2017). Aquaporins in the Skin. Adv. Exp. Med. Biol..

[16] Ghadially R., Brown B.E., Sequeira-Martin S.M., Feingold K.R., Elias P.M. (1995). The aged epidermal permeability barrier. Structural, functional, and lipid biochemical abnormalities in humans and a senescent murine model. J. Clin. Investig..

[17] Ghersetich I., Lotti T., Campanile G., Grappone C., Dini G. (1994). Hyaluronic acid in cutaneous intrinsic aging. Int. J. Dermatol..

[18] Jensen J.M., Forl M., Winoto-Morbach S., Seite S., Schunck M., Proksch E., Schutze S. (2005). Acid and neutral sphingomyelinase, ceramide synthase, and acid ceramidase activities in cutaneous aging. Exp. Dermatol..

[19] Oliver N., Sternlicht M., Gerritsen K., Goldschmeding R. (2010). Could aging human skin use a connective tissue growth factor boost to increase collagen content?. J. Investig. Dermatol..

[20] Silberstein C., Kierbel A., Amodeo G., Zotta E., Bigi F., Berkowski D., Ibarra C. (1999). Functional characterization and localization of AQP3 in the human colon. Braz. J. Med. Biol. Res..

[21] Spector D.A., Wade J.B., Dillow R., Steplock D.A., Weinman E.J. (2002). Expression, localization, and regulation of aquaporin-1 to -3 in rat urothelia. Am. J. Physiol. Ren. Physiol..

[22] Baumgarten R., Van De Pol M.H., Wetzels J.F., Van Os C.H., Deen P.M. (1998). Glycosylation is not essential for vasopressin-dependent routing of aquaporin-2 in transfected Madin-Darby canine kidney cells. J. Am. Soc. Nephrol..

[23] Hendriks G., Koudijs M., van Balkom B.W., Oorschot V., Klumperman J., Deen P.M., van der Sluijs P. (2004). Glycosylation is important for cell surface expression of the water channel aquaporin-2 but is not essential for tetramerization in the endoplasmic reticulum. J. Biol. Chem..

[24] Umenishi F., Narikiyo T., Schrier R.W. (2005). Effect on stability, degradation, expression, and targeting of aquaporin-2 water channel by hyperosmolality in renal epithelial cells. Biochem. Biophys. Res. Commun..

[25] Ferando I., Faas G.C., Mody I. (2016). Diminished KCC2 confounds synapse specificity of LTP during senescence. Nat. Neurosci..

[26] Ikarashi N., Sato W., Toda T., Ishii M., Ochiai W., Sugiyama K. (2012). Inhibitory Effect of Polyphenol-Rich Fraction from the Bark of Acacia mearnsii on Itching Associated with Allergic Dermatitis. Evid Based Complement. Alternat. Med..

[27] Nejsum L.N., Kwon T.H., Jensen U.B., Fumagalli O., Frokiaer J., Krane C.M., Menon A.G., King L.S., Agre P.C., Nielsen S. (2002). Functional requirement of aquaporin-5 in plasma membranes of sweat glands. Proc. Natl. Acad. Sci. USA.

[28] Hara-Chikuma M., Sugiyama Y., Kabashima K., Sohara E., Uchida S., Sasaki S., Inoue S., Miyachi Y. (2012). Involvement of aquaporin-7 in the cutaneous primary immune response through modulation of antigen uptake and migration in dendritic cells. FASEB J..

[29] Karlsson T., Lagerholm B.C., Vikstrom E., Loitto V.M., Magnusson K.E. (2013). Water fluxes through aquaporin-9 prime epithelial cells for rapid wound healing. Biochem. Biophys. Res. Commun..

[30] Loitto V.M., Huang C., Sigal Y.J., Jacobson K. (2007). Filopodia are induced by aquaporin-9 expression. Exp. Cell Res..

[31] Preisser L., Teillet L., Aliotti S., Gobin R., Berthonaud V., Chevalier J., Corman B., Verbavatz J.M. (2000). Downregulation of aquaporin-2 and -3 in aging kidney is independent of V_2_ vasopressin receptor. Am. J. Physiol. Ren. Physiol..

[32] Korlimbinis A., Berry Y., Thibault D., Schey K.L., Truscott R.J. (2009). Protein aging: Truncation of aquaporin 0 in human lens regions is a continuous age-dependent process. Exp. Eye Res..

[33] Bellemere G., Von Stetten O., Oddos T. (2008). Retinoic acid increases aquaporin 3 expression in normal human skin. J. Investig. Dermatol..

[34] Dumas M., Sadick N.S., Noblesse E., Juan M., Lachmann-Weber N., Boury-Jamot M., Sougrat R., Verbavatz J.M., Schnebert S., Bonte F. (2007). Hydrating skin by stimulating biosynthesis of aquaporins. J. Drugs Dermatol..

[35] Jiang Y.J., Kim P., Lu Y.F., Feingold K.R. (2011). PPARγ activators stimulate aquaporin 3 expression in keratinocytes/epidermis. Exp. Dermatol..

